# N-SLEEVE GASTRECTOMY: AN OPTION FOR OBESITY AND GERD

**DOI:** 10.1590/0102-672020190001e1482

**Published:** 2019-12-20

**Authors:** Mariano PALERMO, Edgardo SERRA, Guillermo DUZA

**Affiliations:** 1 Division of Bariatric Surgery, Centro CIEN - DIAGNOMED, Affiliated to the University of Buenos Aires, Buenos Aires, Argentina.

**Keywords:** Sleeve gastrectomy, GERD, Obesity, Bariatric surgery, Gastrectomia vertical, Doença do refluxo gastroesofágico, Cirurgia bariátrica

## Abstract

**Background:**

Obesity represents a growing threat to population health all over the world. Laparoscopic sleeve gastrectomy induces alteration of the esophagogastric angle due to surgery itself, hypotony of the lower esophageal sphincter after division of muscular sling fibers, decrease of the gastric volume and, consequently, increase of intragastric pressure; that’s why some patients have reflux after sleeve.

**Aim:**

To describe a technique and preliminary results of sleeve gastrectomy with a Nissen fundoplication, in order to decrease reflux after sleeve.

**Method:**

In the current article we describe the technique step by step mostly focused on the creation of the wrap and it care.

**Results:**

This procedure was applied in a case of 45 BMI female of 53 years old, with GERD. An endoscopy was done demonstrating a hiatal hernia, and five benign polyps. A Nissen sleeve was performed due to its GERD, hiatal hernia and multiple polyps on the stomach. She tolerated well the procedure and was discharged home uneventfully 48 h after.

**Conclusion:**

N-sleeve is a feasible and safe alternative in obese patients with reflux and hiatal hernia when Roux-en-Y gastric bypass it is not indicated.

## RESUMO

**Racional:**

A obesidade representa ameaça crescente à saúde da população em todo o mundo. A gastrectomia por laparoscopia induz alteração do ângulo esofagogástrico devido à própria técnica, hipotonia do esfíncter esofágico inferior após secção de fibras musculares da junção, diminuição do volume gástrico e, consequentemente, aumento da pressão intragástrica; é por isso que alguns pacientes têm refluxo após a gastrectomia vertical.

**Objetivo:**

Descrever uma técnica e resultados preliminares da gastrectomia vertical com fundoplicatura a Nissen, a fim de diminuir o refluxo após ela.

**Método:**

No artigo atual, descrevemos a técnica passo a passo, principalmente focada na criação da válvula e seu cuidado.

**Resultados:**

Este procedimento foi aplicado em um caso de mulher com IMC 45 de 53 anos com DRGE. Foi realizada endoscopia demonstrando hérnia hiatal e cinco pólipos benignos. A gastrectomia vertical com Nissen foi realizada devido à DRGE, à hérnia hiatal e aos múltiplos pólipos no estômago. Ela tolerou bem o procedimento e recebeu alta sem intercorrências 48 h depois.

**Conclusão:**

A N-gastrectomia vertical (N-sleeve) é alternativa viável e segura em pacientes obesos com refluxo e hérnia hiatal quando não é indicado o desvio gástrico em Y-de-Roux.

## INTRODUCTION

Obesity represents a growing threat to population health all over the world. According to data from the National Health and Nutrition Examination Survey, in 2015/2016, the prevalence of obesity was 39.8% in adults and 18.5% in youth in United States^1^ ^,^ ^2^. Obesity, one of the main factors, is reported to increase the intra-gastric pressure with impaired gastric emptying, the frequency of transient lower esophageal sphincter (LES) relaxation episode and the gastroesophageal pressure gradient, potentially leading to GERD^3^ ^,^ ^4^ ^,^ ^5^ ^,^ ^17^ ^,^ ^18^ ^,^ ^19^. Laparoscopic sleeve gastrectomy induces alteration of the esophagogastric angle due to surgery itself, hypotony of the LES after division of muscular sling fibers, decrease of the gastric volume and, consequently, increase of intragastric pressure, that’s why some patients have reflux after sleeve. 

The objective of this paper was to describe a technique of sleeve gastrectomy with a Nissen fundoplication (described by Prof. Nocca), in order to decrease reflux after sleeve^1^ ^,^ ^2^ ^,^ ^6^ ^,^ ^7^ ^,^ ^8^ ^,^ ^10^ ^,^ ^23^ ^,^ ^24^.

## METHOD

### Surgical technique

**FIGURE 1:**
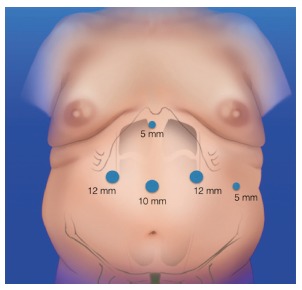
Placement of the trocars

By laparoscopic approach with the surgeon standing between the patient’s legs, five trocars are placed (Figure 1). Pneumoperitoneum is insuflated up to to 15 mmHg. The trocar placement is the same as in standard laparoscopic sleeve gastrectomy (LSG) or Roux-en-Y gastric bypass (RYGBP).

**FIGURE 2:**
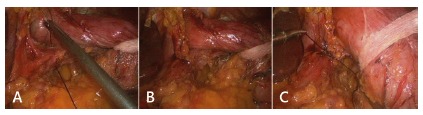
A) Hiatal hernia; B) closure of the hiatal hernia with silk; C) final aspect of the hernia closure calibrating it with a 36 Fr tube

**FIGURE 3:**
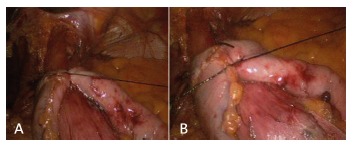
A and B) The wrap is being performed

**FIGURE 4:**
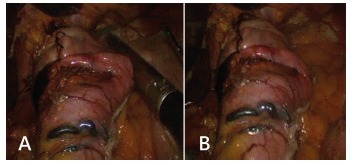
A and B) The last stapling and the final aspect of the N-sleeve

**FIGURE 5:**
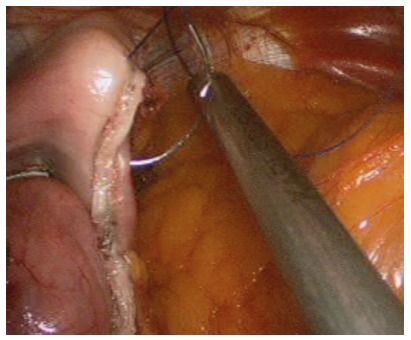
Staple line reinforcement with absorbable sutures

The first step of the N-sleeve is the dissection and reduction of a hiatal hernia. An extension of at least 5 to 6 cm of abdominal esophagus is mobilized and all the anterior and posterior esophageal hiatal space is dissected (Figure 2A). The greater curvature of the stomach is then dissected from the short gastric vessels and gastrocolic ligament, starting 5 cm from the pylorus. Two non-absorbable sutures are used to close the hiatal hernia and a 36 Fr calibration boggie is inserted as a regular LSG (Figures 2B and C). After that step a short 360^º^ valve of 3 cm is created using silk. The wrap valve is fixed to the anterior part of the esophagus (Figure 3A and B). Then the rest of the greater curvature is dissected. A laparoscopic 60 mm linear stapler is used to perform the first division of the antrum. Then the rest of the sleeve gastrectomy is performed as usual with special care in the last fire in order not to cut the “4” layers (Figures 4A and B). All the staple lines are reinforced as we do in the regular LSG (Figure 5). Blue test is performed. We routinely place a JP drain. The postoperative care is the same than the regular LSG.

Contrary to standard LSG, for the N-sleeve some technical details are very important: 1) avoid ischemia of the gastric wall during short gastric vessels dissection; 2) delicate handling of the gastric fundus during fundoplication; 3) avoid double stapling of the gastric fundus^10^ ^,^ ^32^ ^,^ ^36^ ^,^ ^37^.

## RESULTS

This procedure was applied in a female of 53 years; she was admitted to our multidisciplinary group for obesity treatment. She had a BMI of 45. All the preoperative exams were done (laboratory, endoscopy, gastrointestinal series, functional lung test, HPB ultrasound). In the endoscopy a hiatal hernia was demonstrated, and five benign polyps were resected by endoscopy. The patient had GERD. We performed a N-sleeve due to its GERD, hiatal hernia and multiple polyps on the stomach. She tolerated well the procedure, was discharged home uneventfully 48 h after the procedure and in the medium term follow up she did well, with adequate weight loss and non recurrence of her GERD symptoms. We will still follow up her in order to have more long-term data.

**FIGURE 6:**
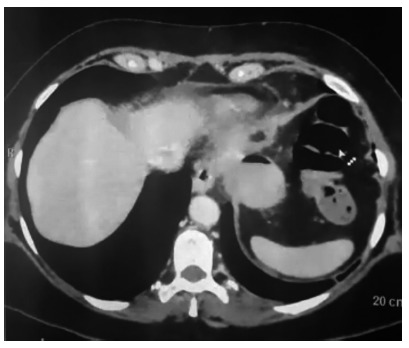
CT demonstrating and adequate Nissen wrap with no complications and a normal sleeve size.

A CT scan was performed for another reason not related with the surgery and we could see the wrap with no complications and an adequate sleeve (Figure 6).

## DISCUSSION

Nocca et al.^23^ by 2016 published the first 25 patients all with esophageal syndromes. Ninety-two had typical symptoms of GERD and two asymptomatic but with esophageal injury. Three months after N-sleeve, 76% of the patients remained asymptomatic without proton pump inhibitor use. At six months and one year, three (12%) patients were still experiencing reflux. Excess weight loss at one year was 58±23%, total weight loss was 27±10%, and body mass index change was -11±4 kg/m². They concluded that N-sleeve seems to be a safe procedure that provides an adequate reflux control with no clear interference on the expected bariatric results of a standard LSG^10^ ^,^ ^12^ ^,^ ^24^.

Regarding GERD, the Montreal conference defines it as a disorder related to reflux of stomach contents leading to discomfort or complications affecting the patient´s quality of life. Typical symptoms are: heartburn (upstream esophageal burning) and regurgitation, and atypical are: epigastric burns, chest pain, respiratory symptoms (chronic cough and asthma), dental erosions^2^ ^,^ ^4^ ^,^ ^20^ ^,^ ^22^ ^,^ ^23^ ^,^ ^26^.

GERD is complex, especially in the era of bariatric surgery^35^. A chronic inflammation can induce more serious lesions, since up to 10-15% of patients develop dysplasia, as Barrett’s esophagus, that can lead to esophageal cancer^9^ ^,^ ^10^ ^,^ ^11^ ^,^ ^13^.

Variables associated with an increased risk of progression of Barrett’s esophagus in dysplasia or adenocarcinoma are: age >70 years, male, absence of treatment with proton pump inhibitor, Barrett’s esophagus longer than 3 cm and esophageal candidiasis^14^ ^,^ ^15^ ^,^ ^16^ ^,^ ^17^.

However, reflux control (by medical treatment or anti-reflux surgery) is associated with regression of Barrett’s mucosa^10^ ^,^ ^12^ ^,^ ^25^ ^,^ ^27^ ^,^ ^28^, an important reason to combine an anti-reflux mechanism to a bariatric procedure.

LSG has evolved into a primary surgical procedure for morbid obesity. It has gained popularity worldwide as a primary bariatric procedure, now established as the most frequent bariatric procedure worldwide^12^ ^,^ ^23^ ^,^ ^34^ ^,^ ^37^. This growth can be explained by several advantages that LSG carries over more complex bariatric procedures, such as RYGBP or duodenal switch, including the absence of most side effects of bypass procedures like dumping syndrome, marginal ulcers, malabsorption, small bowel obstruction and internal hernia, and a better quality of life over gastric banding^17^ ^,^ ^23^ ^,^ ^24^ ^,^ ^32^.

 Besides prior described alterations, LSG decreases ghrelin, hence dismotility^10^ ^,^ ^24^ ^,^ ^25^ ^,^ ^27^. All these factors contribute to expose the patient to the risk of increasing GERD and proton pump inhibitors dependency or developing new GERD onset. On the other hand, weight loss after surgery together with accelerated gastric emptying, decreases acid production and restores esophagogastric angle over time supposing to improve reflux symptoms. However, the presence of preoperative GERD should be considered a relative contraindication to LSG^12^ ^,^ ^24^ ^,^ ^31^.

Here we introduced the concept of N-sleeve as an option to prevent GERD. Although the laparoscopic RYGB was considered the gold standard procedure for obese patients with reflux disease, more than one third of patients who underwent this operation had at least one complication within the 10-year follow-up period^33^. Himpens et al. reported new gastro-esophageal reflux complaints in 21% of patients. Considering all these findings and encouraged by the good results of LSG and concomitant hiatal hernia repair^10^ ^,^ ^12^ ^,^ ^21^ ^,^ ^34^, David Nocca and his team have developed a modification to the usual surgical technique by adding a Nissen fundoplication in order to minimize both leaks and GERD^32^ ^,^ ^37^.

The aim of this article was to describe our N-sleeve technique performed in Buenos Aires as an option for patients who have hiatal hernia with reflux and are not candidates to perform a RYGBP.

## CONCLUSION

N-sleeve is a feasible and safe alternative in obese patients with reflux and hiatal hernia when RYGBP it is not indicated.
